# Insights from yeast into whether the rapamycin inhibition of heat shock transcription factor (Hsf1) can prevent the Hsf1 activation that results from treatment with an Hsp90 inhibitor

**DOI:** 10.18632/oncotarget.2077

**Published:** 2014-06-07

**Authors:** Stefan H. Millson, Peter W. Piper

**Affiliations:** ^1^ Dept. of Molecular Biology and Biotechnology, University of Sheffield, Western Bank, Sheffield, United Kingdom

**Keywords:** heat shock transcription factor, TORC1, Sch9 protein kinase, rapamycin, Hsp90 inhibitor, FKBP12, Ppt1 protein phosphatase, yeast

## Abstract

In human cells TORC1 mTOR (*t*arget *o*f *r*apamycin) protein kinase complex renders heat shock transcription factor 1 (Hsf1) competent for stress activation. In such cells, as well as in yeast, the selective TORC1 inhibitor rapamycin blocks this activation in contrast to Hsp90 inhibitors which potently activate Hsf1. Potentially therefore rapamycin could prevent the Hsf1 activation that frequently compromises the efficiency of Hsp90 inhibitor cancer drugs. Little synergy was found between the effects of rapamycin and the Hsp90 inhibitor radicicol on yeast growth. However certain rapamycin resistance mutations sensitised yeast to Hsp90 inhibitor treatment and an Hsp90 mutation that overactivates Hsf1 sensitised cells to rapamycin. Rapamycin inhibition of the yeast Hsf1 was abolished by this Hsp90 mutation, as well as with the loss of Ppt1, the Hsp90-interacting protein phosphatase that is the ortholog of mammalian PP5. Unexpectedly Hsf1 activation was found to have a requirement for the rapamycin binding immunophilin FKBP12 even in the absence of rapamycin, while TORC1 “bypass” strains revealed that the rapamycin inhibition of yeast Hsf1 is not exerted through two of the major downstream targets of TORC1, the protein phosphatase regulator Tap42 and the protein kinase Sch9 – the latter the ortholog of human S6 protein kinase 1.

*Significance:* A problem with most of the Hsp90 inhibitor drugs now in cancer clinic trials is that they potently activate Hsf1. This leads to an induction of heat shock proteins, many of which have a “pro-survival” role in that they help to protect cells from apopotosis. As the activation of Hsf1 requires TORC1, inhibitors of mTOR kinase could potentially block this activation of Hsf1 and be of value when used in combination drug therapies with Hsp90 inhibitors. However many of the mechanistic details of the TORC1 regulation of Hsf1, as well as the interplay between cellular resistances to rapamycin and to Hsp90 inhibitors, still remain to be resolved.

## INTRODUCTION

### Hsp90, heat shock transcription factor 1 and mTOR kinase – a triage driving cancer progression

Heat shock protein 90 (Hsp90) provides a molecular chaperone function essential for the conformational maturation, activation and maintenance of proteins essential for sustaining all of the hallmarks of cancer. As such it is now a prime target for drug development, with several Hsp90 inhibitors currently in cancer clinic trials [1, 2]. Inhibitors of Hsp90 may prove to be most useful for treating those malignancies where the oncogene driver (e.g. HER2, ALK) has a high dependency on Hsp90 or, in situations such as multiple myeloma, where the buffering of proteotoxic stress is critical for survival [2]. One factor that often compromises the efficiency of these drugs is their tendency to activate heat shock transcription factor 1 (Hsf1). This leads to the induction of heat shock proteins, many of which have a “pro-survival” role in that they help to protect cancer cells from apopotosis [2, 3]. Attention is therefore being directed to developing combination drug therapies that will enable a dual targetting of both Hsf1 and Hsp90 [4]. Like Hsp90, Hsf1 is itself a key permissive factor in cancer progression [5] and overexpressed in certain aggressive cancers (e.g. hepatocellular carcinoma [4]). Furthermore Hsf1 inhibition may not be toxic to normal tissues since Hsf1 knockout mice are viable [5].

A number of small molecule inhibitors of Hsf1 have been identified [6-10]. However the recent discovery that the activation of Hsf1 requires mTOR (*t*arget *o*f *r*apamycin) protein kinase [11] indicates the possibility that TOR kinase inhibitors might be as effective as these inhibitors which act directly on Hsf1 in blocking the Hsf1 activation with by Hsp90 inhibitor drug treatment. Until now, studies that have investigated the combinatorial use of the selective TORC1 inhibitor rapamycin with an Hsp90 inhibitor have not addressed whether the observed effects might be partly due to the rapamycin abrogating the induction of Hsf1 [12-14]. However while this paper was under review, an unbiased drug screen was reported for compounds that could block the Hsf1-directed increases in Hsp70 with Hsp90 inhibition. It revealed that phosphoinositide 3-kinase (PI3K) and mTOR inhibitors could effectively inhibit this increase and potentiate the antitumour efficacy of an Hsp90 inhibitor in many *in vivo* models [15].

As with both Hsp90 and Hsf1, mTOR is often overactivated in cancer; certain gain of function mutations in the mTOR kinase domain being tumorigenic in animal models [16, 17]. This protein kinase forms the catalytic subunit of two distinct multiprotein complexes (TORC1/2), complexes which are central to many of the pathways regulating cell growth and proliferation since they act as the integration “hubs” for diverse signalling inputs [16]. Studies of rapamycin, the natural antibiotic identified as the first highly selective inhibitor of TORC1 (see below), either for treating cancer or to promote a healthier, longer life have been well publicized (especially since this agent has been shown to extend lifespan in flies and mice [18, 19]). Unfortunately the results of the cancer trials of rapamycin and its analogues (rapalogues) have mostly been undistinguished, despite isolated successes. In some cancer cells rapamycin actually promotes oncogenic activity [13], due to an activation of AKT and other signalling molecules of the IGF-1R/IRS-1 signalling system which reflects the loss of a negative feedback regulation on IRS-1 and TORC2 [20, 21]. In addition it can increase NFκB activity and upregulate the expression of IGF-1R and HER2 [22]. Rapamycin also has some undesirable side effects, with low dose, long term treatment inducing insulin resistance [23]. Attention is therefore now being directed to the development of inhibitors that will selectively target the catalytic site of mTOR, drugs that will inhibit both TORC1 and TORC2 [24, 25] (identifier: www.clinicaltrials.gov). There are indications that such drugs might be highly effective when used in combination with Hsp90 inhibitors. Thus both mTOR inhibitors [13] and Hsp90 inhibitors [1, 2] exert potent antiangiogenic activity, with the expectation that improved antiangiogenic therapies may result from a combined use of these agents. The antiangiogenic properties of the TORC1 inhibitor rapamycin are partly attributable to an inhibition of PI3/AKT signalling in endothelial cells, a process strongly activated by vascular endothelial growth factor (VEGF) [26]. The synergism between rapamycin and Hsp90 inhibitors in cultured breast cancer and multiple myeloma has generally been attributed to key downstream targets of IRS-1 and TORC2 signalling being “clients” of Hsp90 [12, 13]. Indeed the rapamycin-promoted oncogenic activity observed in certain tumors employs a number of signaling components highly dependent on Hsp90 (e.g. IGF-1R, IRS-1, HER2, Erk). It should therefore be abrogated by Hsp90 inhibition. However the discovery that the activation of Hsf1 in human cells requires TORC1 [11], opens the possibility that the results of combinatorial usage of rapamycin with an Hsp90 inhibitor may be partly caused by the rapamycin inhibition of TORC1 abolishing the Hsf1 activation with inhibition of Hsp90.

In this study we have employed well-characterised mutant strains of yeast to unravel specific details of the interplay between cellular resistances to rapamycin and an Hsp90 inhibitor; of the TORC1 regulation of Hsf1; of whether the rapamycin inhibition of Hsf1 might be overridden by Hsp90 inhibitor treatment; and of how Hsp90 chaperone system defects might impact on the rapamycin inhibition of Hsf1 activity.

## RESULTS

### Hsp90 inhibitor treatment does not sensitise yeast cells to rapamycin

On the basis of current evidence cellular resistances to rapamycin and to Hsp90 inhibitors might be expected to be, at least to a degree, interdependent. Firstly, both in mammalian systems (see Introduction) and in yeast [27] Hsp90 inhibitors activate Hsf1, whereas rapamycin inhibits the activation of Hsf1 [11](see below). Secondly, the activation of Hsf1 downregulates TORC1 activity and sensitises yeast to rapamycin [28]. Initially therefore we investigated whether there are any synergistic effects between the inhibitory effects of rapamycin and an Hsp90 inhibitor on yeast growth and whether these might be influenced by the loss of the inducible heat shock response.

**Fig 1:**
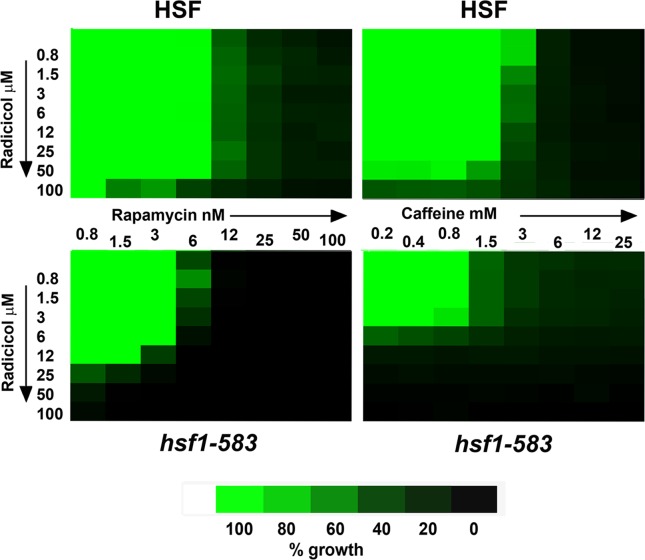
TreeView representation of the growth (46h 28°C) of strains NSY-A (HSF) and NSY-B (*hsf1-583*) in the presence of various combinations of radicicol and rapamycin (left) or radicicol and caffeine (right); the data being represented as a percentage of the growth in the absence of drug.

For this analysis we used two yeast strains (NSY-A, NSY-B; Table 1) which differ in whether they express either a full length or a truncated (residue 1-583) forms of Hsf1. The latter, a form of this transcription factor that lacks the C-terminal activatory domain, provides the Hsf1 function needed for growth to 37°C yet lacks much of the inducible heat shock response [29, 30]. The cells expressing this truncated (1-583) Hsf1 were appreciably more sensitive to the Hsp90 inhibitor radicicol, consistent with their lower levels of Hsp90 [31]. However they were not sensitised to either rapamycin or caffeine (Fig. 1)(the actions of caffeine in yeast having been largely attributed to its inhibition of TORC1 [32]). Importantly, there was very little synergy between the growth inhibitory effects of the Hsp90 inhibitor radicicol and either rapamycin or caffeine, irrespective of whether the cells were expressing the normal full length Hsf1 or the truncated (1-583) Hsf1 (Fig. 1). Therefore, though Hsp90 inhibition normally leads to an activation of Hsf1 and the activation of Hsf1 in the absence of stress has the potential sensitise cells to rapamycin [28], neither the presence nor absence of a stress-activatable Hsf1 appeared to be causing any appreciable synergy between the inhibitory effects of these two drugs, at least in this model system.

**Table 1:** Strains used in this study

Strain	Genotype	Source
W303-1a	MATα; ade2-1 can1-100 his3-11,15 leu2-3,112 trp1-1 ura3-1	Euroscarf
NSY-A	W303-1a hsf1::LEU2 (pRS314-HSF)	[54]
NSY-B	W303-1a hsf1::LEU2 (pRS314-HSF(1-583))	[54]
BY4741	MATa his3-Δ1 leu2-Δ0 met15-Δ ura3-Δ0	Euroscarf
BY4741 fpr1Δ	BY4741 fpr1ΔkanMX4	Research Genetics
BY4741 tor1Δ	BY4741 tor1ΔkanMX4	Research Genetics
BY4741 TOR1.1	BY4741 TOR1.1	This study
BY4741 TOR2.1	BY4741 TOR2.1	This study
BY4741 tor1Δ TOR2.1	BY4741 tor1ΔkanMX4 TOR2.1	This study
BY4741 ppt1Δ	BY4741 ppt1ΔkanMX4	Research Genetics
TB50a	MATa; ura3-52, trp1, leu2, his3, rme1	Robert Loewith
AH308	TB50a SCH9::pRS304	Alexandre Huber
AH333	TB50a sch9^2D3E^::pRS304	Alexandre Huber
AH386	TB50a tip41::hphMX4 SCH9::pRS304	Alexandre Huber
AH387	TB50a tip41::hphMX4 sch9^2D3E^::pRS304	Alexandre Huber
PP30-HSP82	MATa trp1-289, leu2-3,112, his3-200, ura3-52, ade2-101oc, lys2-801am, hsc82::kanMX4, hsp82::kanMX4 [pHSCprom-HSP82 ^a^(LEU2)]	[27]
PP30-hsp82(E381K)	PP30 [pHSCprom-hsp82(E381K) ^a^ (LEU2)]	This study
PP30-HSP82 fpr1Δ	PP30 fpr1ΔhphMX4 [pHSCprom-82 a (LEU2)]	This study
PP30-hsp82(E381K) fpr1Δ	PP30 fpr1ΔhphMX4 [pHSCprom-hsp82(E381K)^a^(2)]	This study
PP30-hsp82(-EEVD)	PP30 [pHSCprom-hsp82(-EEVD) ^a^ (LEU2)]	This study

### Mutations that confer rapamycin resistance in yeast impact on Hsp90 inhibitor sensitivity

**Fig 2:**
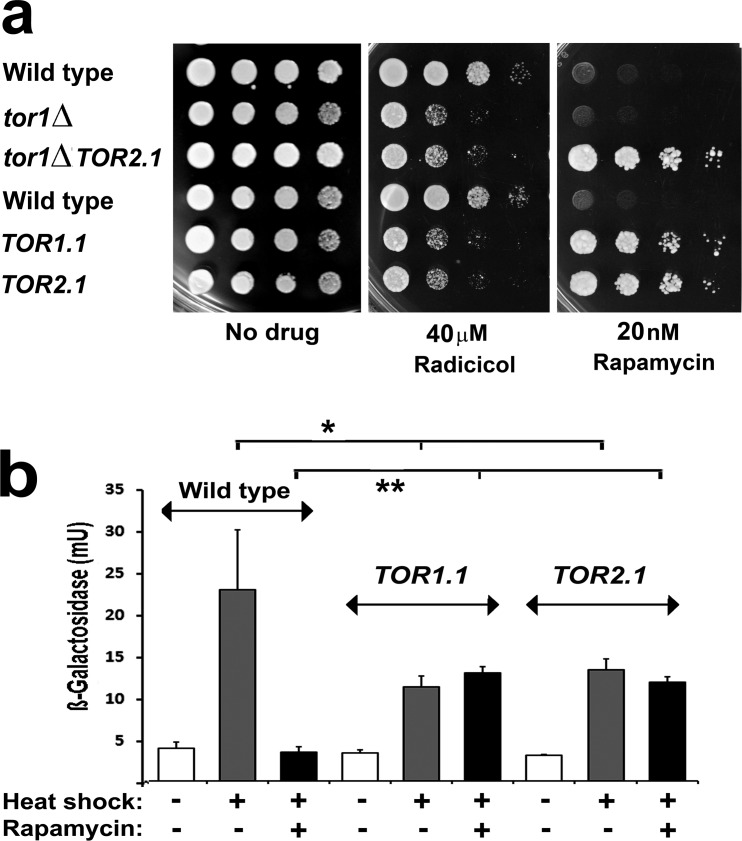
**a:** Serial dilutions of wild type, *tor1Δ*, *TOR1-1, TOR2-1* single mutant and *tor1Δ TOR2-1* double mutant cells of the BY4741 genetic background, pinned onto rich (YPD) medium containing the indicated inhibitor and photographed after 2d growth at 28°C. **b.** Measurements of HSE-*lacZ* reporter gene induction in the wild type*, TOR1*-*1* and *TOR2-1* strains in (a). Cells were treated with or without 100nM rapamycin for 30min at 25°C, HSE-*lacZ* expression being measured after a further 1h at 25°C or following a 25-39°C 1h heat shock (mean and SD of 8 assays on each culture; two-tailed t-test; *=p<0.05; **=p<0.01).

The original genetic identification of TOR kinase as the *t*arget *o*f *r*apamycin came through the analysis of mutations causing rapamycin resistance in yeast [33]. Resistance was generated either through the loss of the immunophilin that binds rapamycin (FKBP12) or with alterations to a conserved serine residue within the *F*KBP12-*r*apamycin complex *b*inding (FRB) domain of either of the two distinct TOR proteins in yeast (S1972R in Tor1 (the *TOR1*-*1 allele)*; S1975I in Tor2 (*TOR2-1*)). Later rapamycin resistance was demonstrated in mammalian cells with the mutation of this conserved serine (S2035) in the single mammalian TOR kinase, mTOR [34, 35]. Not only does the *TOR1.1* mutation generate rapamycin resistance, but it also diminishes Tor kinase activity [36]. Extending these studies in yeast we found that both *TOR1-1* and *TOR2-1* generated a partial sensitivity to radicicol (Fig. 2a), the most potent natural inhibitor of Hsp90 [37].

**Fig 3:**
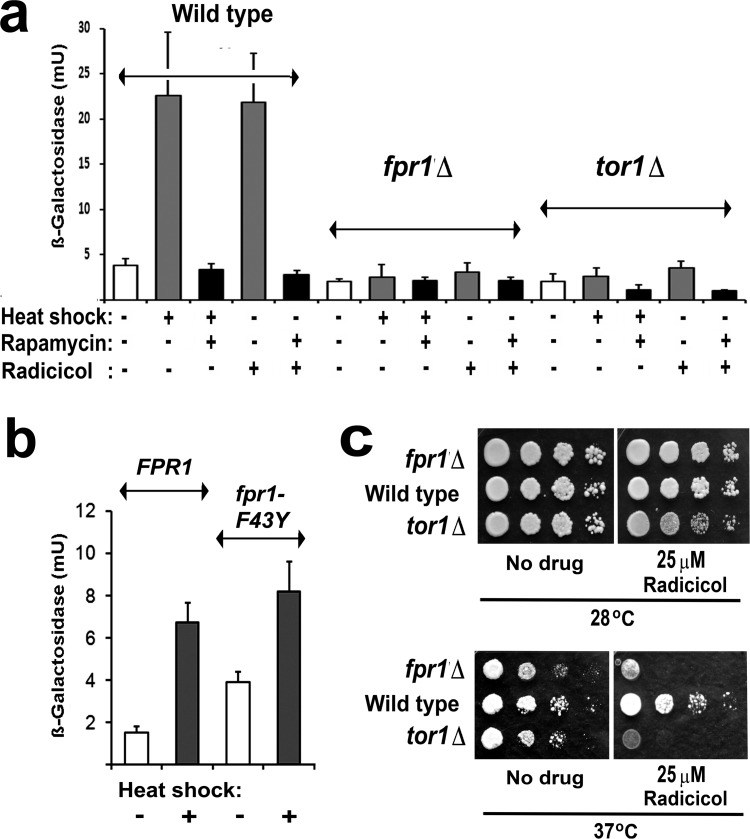
**a:** Measurements of HSE-lacZ induction in wild type, *tor1Δ* and FKBP12-deficient *(fpr1Δ)* cells of the BY4741 genetic background. Cells were treated with or without 100nM rapamycin for 30min at 25°C prior to HSE-*lacZ* induction, either by heat shock (25-39°C for 1h) or the addition of 100μM radicicol (1h 25°C). **b**. The same strains pinned onto rich (YPD) medium with or without radicicol and photographed after 2d growth at either 28°C or 37°C. c. HSE-*lacZ* induction in BY4741*fpr1*Δ mutant cells containing a *LEU2* plasmid borne gene for either the wild type (*FPR1*) or the F43Y mutant (*fpr1- F43Y*) FKBP12.

Whilst assembly of the TORC1/2 complexes needs the Hsp90 chaperone system [38] no evidence has emerged to date to indicate that the enzymatic activities of these complexes are Hsp90-dependent. Of TORC1/2 subunits, only Raptor (Kog1 in yeast) has been shown to interact with Hsp90 [39, 40]. Therefore we investigated whether this effect of *TOR1-1* and *TOR2-1* sensitising cells to Hsp90 inhibitors** (Fig. 2a) might be correlated with an altered regulation of Hsf1. Consistent with this, it was found that these mutations reduced the cellular capacity for induction of a heat shock element (HSE) driven β-galactosidase gene reporter of Hsf1 activity (HSE-lacZ; Fig. 2b). An increased sensitivity to Hsp90 inhibition and dramatically lowered HSE-lacZ induction were also found in the *tor1*Δ mutant (Figs. 2a, 3a,c), a strain which has lowered TORC1 kinase activity due to its lack of the nonessential Tor1 protein (of the two Tor kinase proteins in *Saccharomyces cerevisiae* yeast, Tor1 is nonessential and Tor2 essential, both Tor1 and Tor2 being capable of forming the catalytic subunit of TORC1 whereas TORC2 contains only Tor2).

### Hsf1 activation is lost in yeast, not just with the rapamycin inhibition of TORC1, but also with loss of the rapamycin binding immunophilin FKBP12

The requirement for TORC1 to render Hsf1 competent for stress activation is clearly apparent from the effects of the selective TORC1 inhibitor rapamycin. We found that in yeast, as in human cells [11], administration of this antibiotic rapidly abolished Hsf1 induction (Figs. 2b; 3a,c). Rapamycin must be exerting this effect through its inhibition of TORC1, since it did not inhibit the stress induction of Hsf1 in *TOR1*-*1 and TOR2-1* strains, where the above mutations are causing a genetically dominant loss of the rapamycin inhibition of TORC1 (Fig. 2b).

As described above, rapamycin resistance also arises with the loss of FKBP12, the immunophilin that binds rapamycin. When we investigated HSE-lacZ expression in a strain that lacks this FKBP12 (the *fpr1*Δ mutant; Fig. 3a) we unexpectedly found an almost total absence of stress activation even in the absence of rapamycin. An *FPR1* gene-bearing plasmid was able to restore HSE-lacZ activation in this strain (Fig. 3b). Therefore the stress activation of Hsf1 in yeast has a hitherto undiscovered requirement for FKBP12.

Binding of the inhibitory FKBP12/rapamycin complex to TORC1 is known not to require the peptidyl prolyl isomerise activity of the immunophilin FKBP12 [34]. To analyse whether the activation of Hsf1 has a requirement for this peptidyl prolyl isomerase activity we expressed both wild type and F43Y mutant forms of FKBP12 in *fpr1*Δ cells. F43Y corresponds to a mutation which, in the human FKBP12, lowers the peptidyl prolyl isomerise activity 1000-fold [34]. This F43Y mutation did not abolish the capacity of an introduced *FPR1* gene to rescue stress activation of Hsf1 in *fpr1*Δ cells (Fig. 3b), revealing that FKBP12 does not require its peptidyl prolyl isomerase activity in order to facilitate the activation of Hsf1.

The Hsf1 activation defects of *tor1*Δ and *fpr1*Δ mutant cells (Fig. 3a) are one probable reason why both of these mutants are slightly temperature sensitive (identifier: www.yeastgenome.org/)(Fig. 3c). Only at higher temperatures did the loss of FKBP12 cause an increased cellular sensitivity to the Hsp90 inhibitor radicicol (the *fpr1*Δ mutant, Fig. 3c).

### Rapamycin inhibition of Hsf1 is not exerted through the TORC1 regulation of Tap42 or Sch9, the latter the ortholog of mammalian S6 protein kinase 1

In yeast one can address the issue of whether the TORC1 activation of Hsf1 is direct, or operates through the known signalling events downstream of TORC1. This model organism can be engineered so that each downstream target phosphorylated by TORC1 is rendered independent of TORC1 activity [41]. Yeast TORC1 is known to directly phosphorylate Tap42 (a regulator of PP2A and PP2A-like protein phosphatases [42]), Sch9 (the budding yeast ortholog of the mammalian S6 protein kinase 1 (S6K1) [41]) and Atg13, a subunit of the Atg1 kinase complex. All three targets are also regulated by other protein kinases (Sch9 by Pkh1/2, functional orthologs of mammalian phosphoinositide-dependent protein kinase 1; Atg13 by protein kinase A [43]). Tap42 is rendered constitutively active and independent of upstream signals from TORC1 with the loss of its negative regulator, Tip41 [44]. Sch9, the protein kinase whose inactivation causes most of the rapamycin-induced changes to the yeast phosphoproteome [45], is rendered constitutively active and independent of upstream signals from TORC1 when the amino acids normally phosphorylated by TORC1 at its C-terminus are replaced by acidic residues (the Sch9^2D3E^ allele [41]).

**Fig 4:**
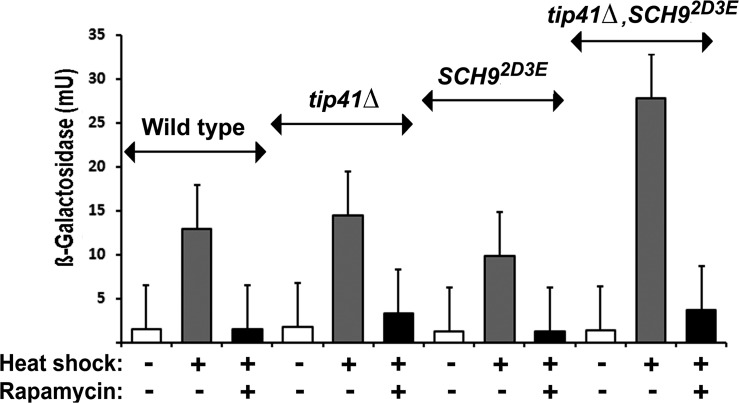
The rapamycin inhibition of HSE-*lacZ* induction is still apparent in *tip41Δ* and ^Sch92D3E^ single mutant, also a tip41Δ, ^Sch92D3E^ double mutant, TORC1 “bypass” strains (cells of the TB50a genetic background; Table 1). The conditions of heat shock and rapamycin treatment were as in Figs. 2,3.

To determine whether the TORC1 regulation of Hsf1 in yeast (Fig. 2b,c) acts through either Tap42 or Sch9 we investigated how rapamycin affects the stress induction of Hsf1 in TORC1 bypass (*tip41Δ*, Sch9^2D3E^ single mutant and *tip41Δ*, Sch9^2D3E^ double mutant) strains (Table 1). We were unable to detect any altered sensitivity to Hsp90 inhibitors in such strains (not shown). Hsf1 induction was still inhibited by rapamycin in *tip41Δ* cells, lacking the TORC1 regulation of Tap42; Sch9^2D3E^ cells lacking the TORC1 regulation of Sch9; as well as in the *tip41Δ*, Sch9^2D3E^ double mutant (Fig. 4). Therefore the Hsf1 inactivation upon rapamycin inhibition of TORC1 (Fig. 2b) does not involve the downregulation of TORC1 signalling through either Tap42 or Sch9. Instead it is possible that TORC1 acts more directly upon Hsf1 in order to render the latter competent for stress activation. It should be noted that a number of the strains studied here (e.g. *TOR1*-*1 and TOR2-1* (Fig. 2b); Sch9^2D3E^ (Fig. 4)) are also defective in the rapamycin-mediated phosphorylation of eIF2α, leading to downregulation of protein synthesis [41]. The rapid rapamycin inhibitions of HSE-lacZ expression, measured as loss of β-galactosidase induction in these experiments (Figs. 2b, 3a,c), are primarily not therefore a reflection of the general downregulation of protein synthesis which normally accompanies the loss of Sch9 activity.

### Rapamycin inhibition of Hsf1 can be lost with mutation of the Hsp90 chaperone system

**Fig 5:**
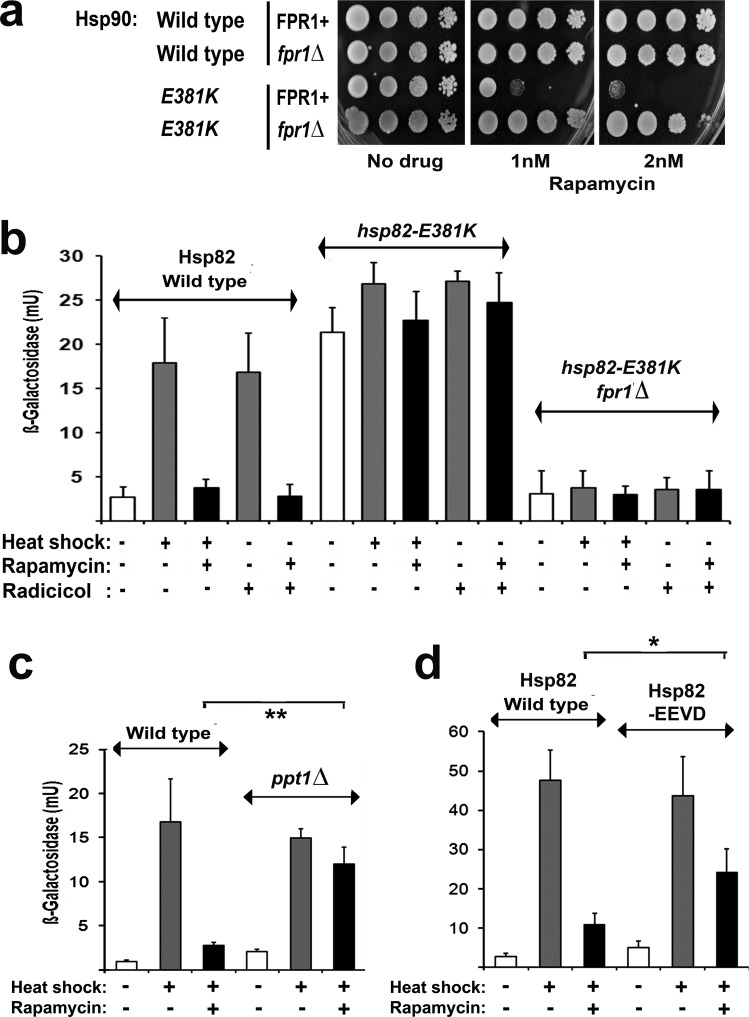
a FPR1+ and *fpr1Δ* cells of the PP30 genetic background expressing either the wild type or the E381K mutant Hsp82 as their sole Hsp90, pinned onto rich (YPD) medium containing the indicated level of rapamycin and photographed after 2d growth at 28°C. b-d Measurements of HSE-*lacZ* reporter gene expression in: (b) wild type, *hsp82-E381K* and *hsp82-E381K fpr1Δ* strains of the PP30 genetic background; (c) *wild type and ppt1Δ* strains of the BY4741 genetic background; and (d) strains of PP30 genetic background expressing Hsp82 the wild type or the –EEVD mutant Hsp82 as their sole Hsp90. The conditions of heat shock and rapamycin treatment were as in Figs. 2,3.

Hsp90 inhibitors potently activate Hsf1, due to the inverse correlation between the activity of Hsf1 and the activity of the Hsp90 chaperone. The resultant induction of antiapoptotic heat shock proteins is one of the main drawbacks to the use of these drugs in cancer therapy (see Introduction). In yeast, los of this Hsp90 suppression of Hsf1 can occur not just through Hsp90 inhibitor treatment [27], but also with mutation to Hsp90 itself (e.g. the hsp82-E381K mutant [27, 46]) and with defects in certain nonessential cochaperones of the Hsp90 chaperone system [47]. Strain PP30-hsp82(E381K)(Table 1) possess a point mutation in its Hsp90 that causes its Hsf1 to be constitutively hyperactive even in the absence of stress [46] (this Hsf1 activation possibly being the reason this strain displays an increased sensitivity to Hsp90 inhibitors [27]). We found that this PP30-hsp82(E381K) also exhibits an extreme, FKBP12 (Fpr1)-dependent hypersensitivity to rapamycin (its growth being inhibited by just 1-2nM rapamycin; Fig. 5a). This sensitisation to rapamycin can be attributed to its high Hsf1 activity, since Hsf1 activation in the absence of heat stress is known to enhance sensitivity to rapamycin [28]. Moreover, unlike the normal Hsf1 activity of PP30-HSP82 cells expressing the wild type Hsp82, this elevated Hsf1 activity of PP30-hsp82(E381K) - though still FKBP12-dependent - was not subject to inhibition by rapamycin (Fig. 5b).

We found that rapamycin also did not inhibit the activation of Hsf1 in cells that lack the serine/threonine protein phosphatase Ppt1 (the *ppt1*Δ mutant, Fig. 5c). Ppt1, the yeast ortholog of mammalian protein phosphatase 5 (PP5), is a dedicated regulator of the Hsp90 chaperone system, its N-terminal tetratricopeptide repeats (TPRs) interacting with the -EEVD motif found at the extreme C-terminus of cytosolic Hsp90s [48]. Finding that this *ppt1*Δ strain displayed a normal HSE-lacZ induction with stress, not inhibited by rapamycin, led us to investigate another strain in which the sole Hsp90 lacks its terminal –EEVD motif (PP30-hsp82(-EEVD); Table 1). In this PP30-hsp82(-EEVD) the HSE-lacZ induction with stress was only partially abolished under our conditions of rapamycin treatment (Fig. 5d). In contrast to *hsp82-E381K* cells, with their high basal HSE-lacZ expression (Fig. 5b), the *ppt1Δ* mutant and the cells in which the Hsp90 lacks the terminal –EEVD displayed essentially unaltered basal levels of HSE-lacZ expression (Fig. 5) as well as sensitivities to radicicol and rapamycin (not shown).

Together these results provide the first demonstration that the rapamycin inhibition of Hsf1 can be lost through Hsp90 mutation or with the loss of an Hsp90 system cochaperone. Rapamycin inhibition of Hsf1 is also lost with rapamycin resistance mutations (Fig. 2b), but these act by abolishing the rapamycin/FKBP12 complex binding to TORC1. In contrast the Hsp90 system defects analysed here do not generate rapamycin resistance (*hsp82-E381K* instead sensitising cells to this antibiotic (Fig. 5a)). It is quite probable therefore that these Hsp90 chaperone defects are preventing the rapamycin inhibition of HSE-lacZ induction at the level of the Hsp90 suppression of Hsf1 activity.

## DISCUSSION

It is important to develop strategies for the inhibition of Hsf1, since this transcription factor is a driver of cancer progression and the Hsf1-directed heat shock response frequently causes a degree of resistance to Hsp90 inhibitor cancer drugs (see Introduction). This study has exploited well characterised mutants of yeast to investigate the rapamycin inhibition of the native Hsf1 in this model organism; as well as how this is inhibition might be affected by the inhibition and mutation of the Hsp90 chaperone machine. Both in yeast and in human cells rapamycin and Hsp90 inhibitors have potent, yet opposing, effects on the activity of Hsf1. Rapamycin is an inhibitor of Hsf1 activity (Fig. 2b) [11], while Hsp90 inhibitors are activators of Hsf1. The issue therefore arises of what happens to both growth inhibition and Hsf1 activity when rapamycin is used in combination with an Hsp90 inhibitor.

In this study we found little synergy between the inhibitory effects of rapamycin and the Hsp90 inhibitor radicicol on the growth of yeast, irrespective of whether the cells possessed a stress-inducible form of Hsf1 (Fig. 1). This may be due to an overriding effect of rapamycin, which rapidly blocked any Hsf1 induction by the Hsp90 inhibitor (Fig. 3a). Such findings contrast with those of a recent study in which mTOR inhibitors were found to potentiate the efficacy of the Hsp90 inhibitor ganetespib in several tumor cell lines [15]. Such differences - yeast versus tumor cells - are perhaps to be expected, especially considering the considerably less complex role of TORC1 in yeast as compared to mammalian systems.

The novelty of this work is that it has provided the first demonstration that Hsp90 inhibitor resistance can be influenced by mutations causing resistance to rapamycin (Fig. 2a) and, conversely, that rapamycin resistance can be influenced by mutations in the Hsp90 chaperone machine (Fig. 5a). While rapamycin inhibits the activation of Hsf1, it is without any appreciable effect on immediate HSE-lacZ expression in cells that substantially lack an inducible Hsf1 (e.g. *tor1Δ*, *fpr1Δ*; Fig. 3a). In yeast this inhibition of Hsf1 is not exerted through either Tap42 or Sch9, major downstream targets phosphorylated by TORC1 (Fig. 4). It may therefore reflect a more direct TORC1 activation of Hsf1. In human cells Hsf1 activation is known to require its TORC1-dependent phosphorylation of serine 326 [11]. The phosphorylation of this serine residue is attracting increased attention as a marker of the highly malignant state [49].

This study found that the *TOR1.1, TOR2.1* and *fpr1*Δ rapamycin resistance mutations sensitise cells to Hsp90 inhibitor treatment (Fig. 2a, 3c) and abrogate the inducible heat shock response even in the absence of rapamycin (Fig. 2b,3a); that the rapamycin inhibition of Hsf1 activation does not involve the downstream TORC1 regulation of Tap42 or Sch9; also that the rapamycin inhibition of Hsf1 can be overridden by Hsp90 mutation and with loss of an Hsp90 system cochaperone (Fig. 5). Notably we find that Hsf1 activation has a requirement for the rapamycin-binding immunophilin FKBP12, though not the peptidyl prolyl isomerise activity of this FKBP12 (Fig. 3). It will be interesting to discover if the Hsf1 activation in human cells has a similar requirement for the conserved immunophilin FKBP12.

In mammalian systems, the signalling downstream of TORC1 is considerably more complex than in yeast. However much of it still operates through S6K1 [16]. An extension of these yeast genetic studies therefore has the potential to reveal yet more details of how TORC1 regulates Hsf1, how the inhibition of TORC1 acts to suppress the inducible heat shock response and how this inhibition, in turn, impacts on the Hsp90 suppression of Hsf1 activity. Many of the mechanisms uncovered this way are likely to be conserved, yeast to man. Furthermore the human Hsf1 can, by mutation, be rendered functional in yeast whereupon much of its regulation is found to be conserved in this model eukaryote [50, 51].

In aging cells and tissues the levels of TORC1 activity are one of the factors driving senescence, with the result that decreases in this activity have the potential to slow aging [52]. In cancer drug therapy the aim is generally a downregulation of mTOR and Hsf1, whereas in healthy aging an increased Hsf1 activity should potentially be beneficial in that it will increase chaperone levels, thereby causing the improved proteostasis that could counteract sarcopenia and neurodegenerative disease [3, 10]. This study has found that the TORC1 regulation of the transient heat shock response of vegetative yeast occurs independently of Sch9 (ortholog of S6K1; Fig. 4). However a decreased activity of S6K, yet an increased activity of Hsf1 are increasingly being identified as two factors important for extending the chronological lifespan of model organisms [18, 19]. They are also - at least to a degree - interdependent, Hsf1 being essential for the extension to lifespan when S6K is downregulated in aging *Caenorhabditis elegans* [53]. Future studies will therefore need to address the issue of whether the TORC1 regulation of Hsf1, studied here in the inducible heat shock response of vegetative yeast cells, differs from how TORC1 might regulate Hsf1 in the normal processes of aging.

## MATERIALS AND METHODS

Yeast strains used in this study are listed in Table 1. These were routinely maintained on YPD (2% (w/v) bactopeptone, 1% yeast extract, 2% glucose) 1.5% agar plates. Transformations with a *URA3* vector bearing a LacZ gene under heat shock element control (HSE-LacZ) and measurement of levels of β-galactosidase expression were as previously described [27]. Cultures containing this plasmid were grown on standard defined (SD) minus uracil dropout medium and subjected to stress and/or antibiotic treatment as described in the figure legends. For the study in Fig. 3c strain BY4741 *fpr1Δ* containing this HSE-LacZ vector was transformed with additional YCplac111-based *LEU2* vectors bearing either the wild type or F43Y mutant forms of the *FPR1* gene under native promoter control, then grown on SD minus uracil and leucine prior to measurements of their HSE-LacZ expression.

For drug sensitivity assays, overnight YPD cultures were either serially diluted, then pinned onto YPD agar containing the indicated antibiotic and the plates incubated as indicated in the figure legends; or they were inoculated into complete SD medium (1×10^5^ cells/ml), this dilution being used to set up radicicol and rapamycin or radicicol and caffeine dose response matrixes (final volume 0.2 ml/well) in 96-well microtiter plates. After 46h growth at 28°C the cells were re-suspended by agitation, their absorbance determined at 595 nm with a Multiskan Ascent platereader with correction for the background from the corresponding medium, and the data quantitatively displayed with color using the program Java Treeview 1.1.3 (http://jtreeview.sourceforge.net/).
